# Draft Genome Sequence of *Escherichia coli* K-12 (ATCC 29425)

**DOI:** 10.1128/genomeA.00574-17

**Published:** 2017-07-06

**Authors:** Kathleen C. Engelbrecht, Catherine Putonti, David W. Koenig, Alan J. Wolfe

**Affiliations:** a Corporate Research and Engineering, Kimberly-Clark Corporation, Neenah, Wisconsin, USA; b Bioinformatics Program, Loyola University Chicago, Chicago, Illinois, USA; c Department of Biology, Loyola University Chicago, Chicago, Illinois, USA; d Department of Computer Science, Loyola University Chicago, Chicago, Illinois, USA; e Department of Microbiology and Immunology, Stritch School of Medicine, Health Sciences Division, Loyola University Chicago, Maywood, Illinois, USA

## Abstract

A draft genome sequence for *Escherichia coli* ATCC 29425 was investigated. The size of the genome was 4,608,319 bp, with an observed G+C content of 50.68%. This assembly consisted of 80 contigs, with an average coverage of 122.2×, including one contig representative of the complete genome for the temperate phage P1.

## GENOME ANNOUNCEMENT

*Escherichia coli* K-12 strains are widely used in molecular biology. Here, we present the genome sequence of *E. coli* strain ATCC 29425 ([Migula] Castellani and Chalmers) to add to the understanding of the diversity of this species.

The strain was purchased from ATCC and grown on 5% sheep blood agar (BD BBL prepared plated medium) under 5% CO_2_ at 35°C for 48 h. The cells were resuspended in 0.5 ml of DNA extraction buffer (20 mM Tris-HCl, 2 mM EDTA, 1.2% Triton X-100 [pH 8]), followed by the addition of 50 μl of lysozyme (20 mg/ml), 30 μl of mutanolysin, and 5 μl of RNase (10 mg/ml). After a 1-h incubation at 37°C, 80 μl of 10% SDS and 20 μl of proteinase K were added, followed by a 2-h incubation at 55°C. Two hundred ten microliters of 6 M NaCl and 700 μl of phenol-chloroform were added. After a 30-min incubation with rotation, the solutions were centrifuged at 13,500 rpm for 10 min, and the aqueous phase was extracted. An equivalent volume of isopropanol was added, and the solution was centrifuged at 13,500 rpm for 10 min after a 10-min incubation. The supernatant was decanted and the DNA pellet precipitated using 600 μl of 70% ethanol. Following ethanol evaporation, the DNA pellet was resuspended in Tris-EDTA and stored at −20°C.

Genomic DNA was diluted in water to a concentration of 0.2 ng/μl, as measured by a fluorometric-based method (Life Technologies, Inc.). Library preparation of 5 μl (1 ng) of the isolated DNA was performed using the Nextera XT DNA library preparation kit. The library was sequenced on the MiSeq sequencer (Illumina) using the MiSeq reagent kit version 2 (500 cycles). The run produced 1,257,935 paired-end reads. Reads were trimmed, removing adapter sequences and phiX contaminants, by the BBDuk program from the BBMap package (http://sourceforge.net/projects/bbmap/). The trimmed reads were then assembled using SPAdes (version 3.5) (1) and scaffolded by SSPACE (2). Eighty contigs, varying in size from 466 bp to 368,540 bp, were produced, with an average coverage of 122.2× and an *N*_50_ value of 157,792 bp. The genome size was 4,608,319 bp, with an observed G+C content of 50.68%. Each contig sequence was next queried via BLASTn queries to the nr/nt database. One contig, deposited as pATCC29425, showed the greatest homology to the complete genome of the temperate phage P1 (query coverage, 98%; sequence identity, 99%; GenBank accession no. AF234172.1) and other *Enterobacteriaceae* plasmid sequences. Genome annotations were produced using the software tool Peasant (3). Eight rRNA genes, 82 tRNA genes, and 4,450 protein-coding sequences were detected. Two clustered regularly interspaced short palindromic repeat (CRISPR) arrays were found (4). These two arrays are highly conserved among *E. coli* K-12 genomes, exhibiting 100% sequence identity and coverage to sequences in the nr/nt BLAST database.

### Accession number(s).

The draft whole-genome project for *E. coli* ATCC 29425 has been deposited at DDBJ/EMBL/GenBank under accession number NARF00000000. Raw sequence reads are deposited at DDBJ/EMBL/GenBank under accession number SRR5364281.
